# A genome-scale CRISPR-Cas9 screening method for protein stability reveals novel regulators of Cdc25A

**DOI:** 10.1038/celldisc.2016.14

**Published:** 2016-05-24

**Authors:** Yuanzhong Wu, Liwen Zhou, Xin Wang, Jinping Lu, Ruhua Zhang, Xiaoting Liang, Li Wang, Wuguo Deng, Yi-Xin Zeng, Haojie Huang, Tiebang Kang

**Affiliations:** 1 Sun Yat-sen University Cancer Center, State Key Laboratory of Oncology in South China, Collaborative Innovation Center for Cancer Medicine, Guangzhou, China; 2 Clinical Laboratory and Medical Research Center, Zhuhai Hospital, Jinan University, Zhuhai People's Hospital, Zhuhai, China; 3 Department of Biochemistry and Molecular Biology, Mayo Clinic College of Medicine, Rochester, MN, USA; 4 Guangdong Provincial Key Laboratory of Tumor Targeted Drugs, Guangzhou Doublle Bioproducts Co. Ltd., Guangzhou, China

**Keywords:** acetylation, Cdc25A, CRISPR-Cas9 screening, protein stability, ubiquitination

## Abstract

The regulation of stability is particularly crucial for unstable proteins in cells. However, a convenient and unbiased method of identifying regulators of protein stability remains to be developed. Recently, a genome-scale CRISPR-Cas9 library has been established as a genetic tool to mediate loss-of-function screening. Here, we developed a protein stability regulators screening assay (Pro-SRSA) by combining the whole-genome CRISPR-Cas9 library with a dual-fluorescence-based protein stability reporter and high-throughput sequencing to screen for regulators of protein stability. Using Cdc25A as an example, Cul4B-DDB1^DCAF8^ was identified as a new E3 ligase for Cdc25A. Moreover, the acetylation of Cdc25A at lysine 150, which was acetylated by p300/CBP and deacetylated by HDAC3, prevented the ubiquitin-mediated degradation of Cdc25A by the proteasome. This is the first study to report that acetylation, as a novel posttranslational modification, modulates Cdc25A stability, and we suggest that this unbiased CRISPR-Cas9 screening method at the genome scale may be widely used to globally identify regulators of protein stability.

## Introduction

Fine-tuned degradation of proteins has a crucial role in cell cycle progression, cell growth, differentiation and signaling transduction under a variety of physiological conditions [1, 2]. The inappropriate degradation of some cancer-related proteins may induce cellular transformation, whereas the dysregulation of many other proteins is related to other diseases [2, 3]. Thus, understanding the precise regulation of protein degradation is an important issue for not only understanding biological mechanisms but also for developing therapeutic interventions for diseases [4, 5]. However, the unbiased and global delineation of the regulators of a protein homeostasis remains challenging because of the lack of an efficient method.

Thus far, there are multiple methods that can be used to identify key regulators of protein stability in mammalian cells, with each having obvious disadvantages. Co-immunoprecipitation purification combined with mass spectrometry identification is the most common method [6, 7]. However, this method is only suitable for proteins with strong interactions, thus it is easy to miss the regulators whose interactions are weak or transient [8–10]. The other method involves screening with small libraries of small interfering RNA (siRNA), which is coupled with western blotting or immunofluorescence analysis to monitor the protein expression level [11–13]. However, for most laboratories, this method is restricted to a low-throughput screening because high-throughput or whole-genome screening is time consuming, labor intensive and expensive. Another method is motif prediction, which is based on the knowledge that many enzymes such as E3 ligases and kinases share conserved modification motifs and affect the target homeostasis [14–16]. However, the motif is not a stringent specification and often randomly occurs in the protein sequence, and many different enzymes may share the same motif. Therefore, establishing a highly efficient and unbiased method to globally screen for regulators of protein stability is urgent and necessary.

A fluorescence-based system to monitor protein dynamics at the single-cell level has emerged recently. Among the different techniques, global protein stability (GPS) analysis and protein turnover assay (ProTA) have been demonstrated to work well [17–19]. Both methods are based on dual fluorescence, with one fused to the protein of interest and another as the reference. In the GPS system [17, 18], a cassette containing DsRed-IRES-EGFP (enhanced green fluorescent protein)-X was used to reflect the stability of protein X. The internal ribosome entry site (IRES) permits translation of both DsRed and EGFP-X fusion proteins at a certain ratio. In the ProTA system [19], X-mEGFP-Ub_k0_-mRFP was applied as a reporter, which allowed for the translation of a fusion protein from one open reading frame. In this system, the Ub_k0_ between monomeric forms of EGFP and RFP (mEGFP and mRFP) would warrant efficient cleavage after the Gly76 residue in the ubiquitin moiety to generate the X-mEGFP fusion protein and mRFP, which also results in a certain ratio of X-mEGFP/mRFP fluorescence intensity. In both GPS and ProTA systems, any event that affects the X protein stability would change the ratio of EGFP/DsRed, which would be analyzed or monitored by flow cytometry.

Several groups have recently shown that the CRISPR-Cas9 library is an easy and efficient system for performing genome-scale loss-of-function screening [20–23]. Compared with short hairpin RNA targeting of mRNA for knockdown [24], the CRISPR-Cas9 introduces indel mutations into the genomic DNA to mediate gene knockout. Thus, homozygous knockout confers high sensitivity for the screening, whereas incomplete knockdown retains gene function. The CRISPR-Cas9 library shows a high validation rate for top screening hits, and the off-target effect does not appear to seriously hamper the genome-scale screening [21, 22]. In this report, we sought to explore the feasibility of using the CRISPR-Cas9 knockout library to globally screen for stability regulators of any unstable protein.

## Results

### The pAd-DsRed-IRES-EGFP-X reporter couples with CRISPR-Cas9 gene knockout to determine X protein stability

**Figure 1:**
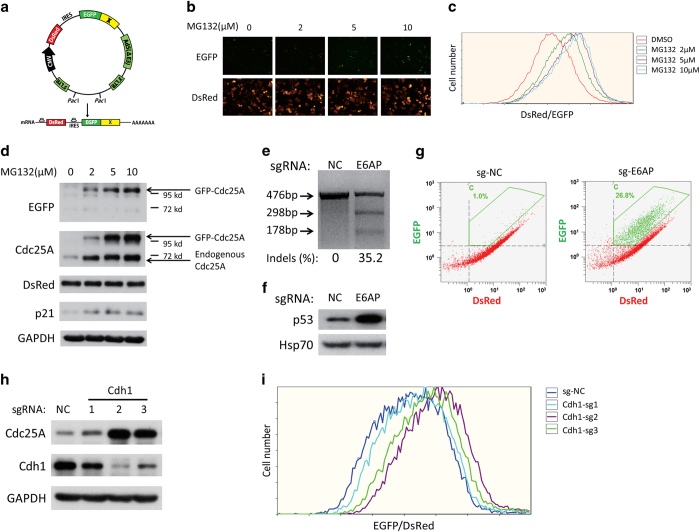
Determination of protein stability by the pAd-DsRed-IRES-EGFP-X reporter system coupled with CRISPR-Cas9 gene knockout. (**a**) Schematic for the pAd-DsRed-IRES-EGFP-X (protein) reporter system. The DsRed-IRES-EGFP-X element was cloned into an adenovirus vector. (**b, c, d**) HeLa cells were infected with pAd-DsRed-IRES-EGFP-Cdc25A for 48 h, and then treated with the indicated concentrations of MG132 for 2 h. The protein signals were analyzed by fluorescence (**b**), flow cytometry (**c**) and western blotting (**d**). (**e, f**) HeLa cells infected with lenti-CRISPR sgRNA targeting E6AP or control were incubated with puromycin (0.5 μg ml^−1^) for 7 days. Then, the indels in the cells were analyzed by T7E1 digestion (**e**), and the endogenous p53 protein was detected using western blotting analysis (**f**). (**g**) Stable HeLa cells in (**e**) were re-infected with pAd-DsRed-IRES-EGFP-p53 adenovirus for 48 h and then analyzed by flow cytometry. (**h, i**) HeLa cells infected with lenti-CRISPR sgRNAs targeting Cdh1 or control were incubated with puromycin (0.5 μg ml^−1^) for 7 days, and endogenous Cdc25A and Cdh1 were analyzed by western blotting (**h**), or re-infected with pAd-DsRed-IRES-EGFP-Cdc25A adenovirus for 48 h, and then analyzed by flow cytometry (**i**).

To establish a reporter to monitor protein stability in live cells, we developed an adenovirus reporter vector named pAd-DsRed-IRES-EGFP-X based on the retroviral reporter vector pCMV-DsRed-IRES-EGFP-X in the GPS system (Figure 1a). There are many advantages for adenovirus-based infection. For instance, adenovirus does not insert into the genome, thereby avoiding the perturbation of endogenous genes; adenovirus can reach high infection efficiency without drug selection. Then, we used Cdc25A as an example to evaluate feasibility of the adenovirus reporter to monitor protein stability in live cells. The pAd-DsRed-IRES-EGFP-Cdc25A reporter was generated using this adenovirus vector cloned with the Cdc25A ORF, and it easily reached >95% infection efficiency in HeLa cells (Supplementary Figure S1). More importantly, as shown in Figure 1b–d, the increase in both EGFP-Cdc25A signal and endogenous Cdc25A stability positively correlated with the concentrations of MG132, a proteasome inhibitor, whereas the DsRed signal remained constant, demonstrating that the EGFP/DsRed ratio is able to reflect Cdc25A stability in cells, which indicated that cells with altered EGFP/DsRed ratios may be sorted by flow cytometry.

Then, we validated whether the knockout of upstream regulators through CRISPR-Cas9 system could induce the alteration of target protein-EGFP/DsRed ratio. Here we chose p53 as an example using HeLa cells, which is positive for human papillomavirus, and E6 oncoprotein of human papillomavirus will assist human E6AP (E6-associated protein) to maintain extremely low levels of p53 protein in this cell line [25]. We used a lenti-CRISPR-Cas9 plasmid targeting E6AP to infect HeLa cells. After 7 days of infection, 35.2% of E6AP underwent indels as shown by the Surveyor assay (Figure 1e), and endogenous p53 was markedly stabilized (Figure 1f). Stable sg-E6AP-infected HeLa cells were re-infected with the pAd-DsRed-IRES-EGFP-p53 virus for 48 h. As shown in Figure 1g, a large population of cells with an enhancement of the EGFP/DsRed ratio was clearly detected by flow cytometry, and the cells with an altered ratio could be easily sorted later by flow cytometry. In addition, Cdc25A was chosen as another example of unstable proteins using three single-guide RNA (sgRNA) lenti-CRISPR-Cas9 plasmids against Cdh1, a known negative regulator of Cdc25A [26]. As shown in Figure 1h and i, the sgRNAs knockdown efficiency of Cdh1 was positively correlated with the stabilization of Cdc25A using both western blot and flow cytometry analysis. Collectively, these results show that combining the CRISPR-Cas9 knockout library with the pAd-DsRed-IRES-EGFP-X reporter system is feasible for establishing the genome-scale protein stability regulators screening assay (Pro-SRSA).

### A genome-scale screening identifies the negative regulators of Cdc25A stability using the Pro-SRSA

**Figure 2:**
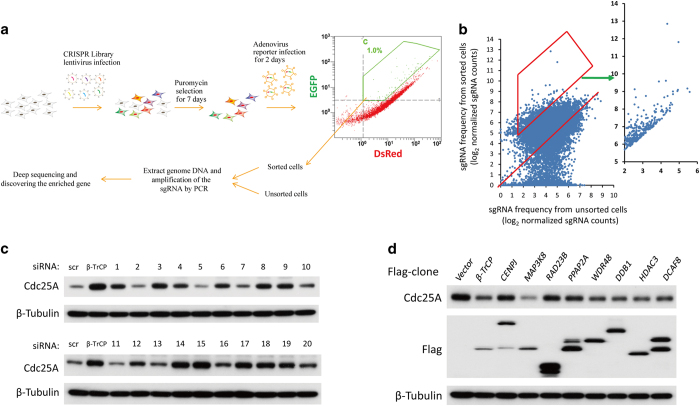
Establishment of Pro-SRSA and screening for regulators of Cdc25A stability. (**a**) The workflow for Pro-SRSA. (**b**) Using Cdc25A as a target of Pro-SRSA, a scatter plot shows the enrichment of sgRNAs after flow cytometric sorting and high-throughput sequencing. (**c**) HeLa cells were transfected with the indicated siRNA for 48 h, and were analyzed using western blotting, the numbers refer to Table 1. (**d**) HEK293T cells transfected with the indicated Flag-tagged proteins for 48 h were analyzed using western blot.

The Pro-SRSA is outlined in Figure 2a. The CRISPR-Cas9 library we used was the GeCKO library, which contains 18 080 genes with 64 751 unique guide sequences to target the whole genome and has been demonstrated to have good performance in genome-scale screening [21]. Once again, we used Cdc25A as a target. Briefly, HeLa cells were infected with the CRISPR-Cas9 library at 0.2 multiplicity of infection (MOI) to control only one transgene copy number in most of the cells and selected by puromycin, and then re-infected with the pAd-DsRed-IRES-EGFP-Cdc25A adenovirus. Up to 48 h post-reporter infection, the cell population in the top 1% of the ratio of EGFP/DsRed was sorted out for the high-throughput sequencing of the sgRNAs distributions, compared with those in the unsorted control cells, we could calculate the enrichment for each sgRNA (Figure 2b, Supplementary Table S1). Given that Cdc25A is regulated during the cell cycle, it is possible that the cell cycle distribution may be different between the sorted cells and the unsorted cells. To rule out this possibility, the cell cycle distributions were monitored and the cell cycle profile was marginally altered in the sorted cells compared with that in the unsorted cells (Supplementary Figure S2). By analyzing the enrichment of sgRNAs, we could determine which knockout contributed to the stabilization of Cdc25A. Compared with the unsorted total cells, many highly enriched genes were identified in the sorted populations, including several genes related to the proteasome pathway: ANAPC15, USP47, PSMF1, PSMB9, PSMG4, PSMD9 and PSMB4 were identified (Supplementary Table S2). ANAPC15, which is a component of APC/C^Cdh1^ E3 ligase, and USP47, which is an interacting protein of SCF^β-TrCP^, have been reported to downregulate Cdc25A [27, 28], indicating that this method is able to identify regulators of Cdc25A stability.

Next, 20 genes that were enriched by approximately 10-fold or above were chosen for validation (Table 1, Supplementary Table S2), and a small siRNA library with three unique target sequences per gene was transfected as a mix to ensure its knockdown efficiency in cells. Excitingly, knockdown of 60% of the genes (12 of 20), that is, ZGPAT, MAP3K8, PPAP2A, WDR48, USP47, HDAC3, DDB1, DCAF8, RNF13, CENPJ, HES3 and RAD23B, was shown to clearly stabilize Cdc25A in cells (Figure 2c), whereas the ectopic expression of 5 of 8 of these genes, that is, MAP3K8, WDR48, HDAC3, DDB1 and DCAF8, downregulated endogenous Cdc25A in cells (Figure 2d). Among these five genes, DDB1, DCAF8 (DDB1 and Cul4 associated factor 8) and HDAC3, were chosen for further investigation in this study.

### Cul4B-DDB1^DCAF8^ is a new E3 ligase of Cdc25A

**Figure 3:**
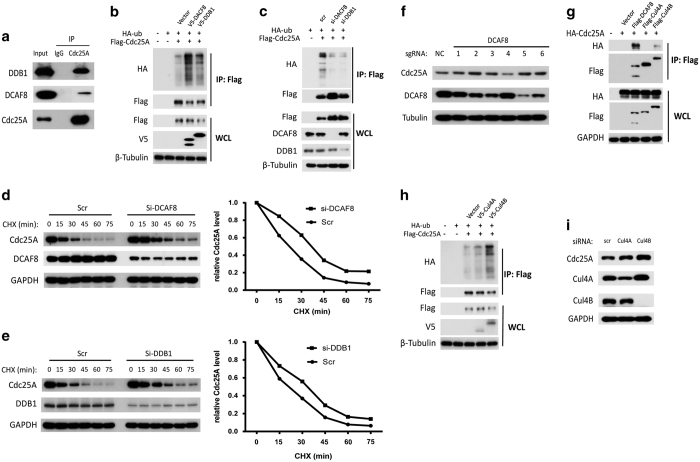
Cul4B-DDB1^DCAF8^degrades Cdc25A. (**a**) The endogenous interaction between Cdc25A and DDB1 or DCAF8. Immunoprecipitation (IP) was carried out using HEK293T lysates. (**b**) Overexpression of DCAF8 or DDB1 enhanced Cdc25A ubiquitination. HEK293T cells transfected with the indicated plasmids for 46 h were incubated with MG132 (10 μM) for 2 h, then were lysed and subjected to IP and western blot analysis. (**c**) Knockdown of DCAF8 or DDB1 decreased Cdc25A ubiquitination. HEK293T cells transfected with the indicated siRNA for 24 h were transfected with the indicated plasmids for another 22 h, and were incubated with MG132 (10 μM) for 2 h, then were lysed and subjected to IP and western blot analysis. (**d, e**) HEK293T cells transfected with DCAF8 or DDB1 siRNAs for 48 h were treated with cycloheximide (CHX, 20 μg ml^−1^) for the indicated time, and endogenous Cdc25A protein levels were analyzed. (**f**) HeLa cells infected with each one of the lenti-CRISPR sgRNAs targeting DCAF8 as indicated or control were incubated with puromycin (0.5 μg ml^−1^) for 7 days, and were analyzed by western blot. (**g**) Interaction between Cdc25A and Cul4B. HEK293T cells transfected with the indicated plasmids for 46 h were incubated with MG132 (10 μM) for 2 h, and were subjected to IP using anti-Flag antibody, which was followed by western blot. (**h**) Cul4B promoted ubiquitination of Cdc25A. HEK293T cells transfected with the indicated plasmids for 46 h were incubated with MG132 (10 μM) for 2 h, and were lysed and subjected to IP and western blot. (**i**) Knockdown of Cul4B stabilized Cdc25A. HeLa cells transfected with the indicated siRNA for 48 h were analyzed using western blot.

Given that Cul4-DDB1^DCAFs^ is an E3 ligase responsible for degradation of some substrates [29], and both DCAF8 and DDB1 were hits in our screening and were also validated using both siRNA and ectopic expression (Figure 2c and d), we surmised that the Cul4-DDB1^DCAF8^ complex may be an E3 ligase that is capable of degrading Cdc25A, similar to APC/C^Cdh1^ and SCF^β-TrCP^. First, the interaction between Cdc25A and DCAF8 or DDB1 was detected at both endogenous and exogenous levels in cells (Figure 3a, Supplementary Figure S3). Second, Cdc25A ubiquitination was increased in cells overexpressing DCAF8 or DDB1 (Figure 3b), whereas knockdown of DCAF8 or DDB1 decreased the ubiquitination of Cdc25A (Figure 3c). Third, the half-life of Cdc25A was prolonged under the knockdown of either DCAF8 or DDB1 (Figure 3d and e). To further validate the effect of DCAF8 on Cdc25A, we cloned all six target sequences of DCAF8 in the GeCKO library and tested their effects on Cdc25A. As shown in Figure 3f, the sgRNA-2, 3, 5 and 6 were better than sgRNA-1 and 4 in both the knockdown efficiency for DCAF8 and the Cdc25A stabilization. Consistently, listed in the Supplementary Table S1, the sgRNA-2, 3 and 6 in the sorted cells were enriched for 10.70-, 7.29- and 3.86-fold compared with the unsorted cells, respectively. Cul4 contains two homolog members, Cul4A and Cul4B, and both can form the E3 ligase with DDB1 and DCAFs [29]. However, the interaction between Cdc25A and Cul4B, but not Cul4A, was detected (Figure 3g). Overexpression of Cul4B markedly enhanced the ubiquitination of Cdc25A, whereas Cul4A only showed marginal effects on Cdc25A (Figure 3h). Consistently, Cdc25A was also more stabilized in cells with a knockdown of Cul4B compared with those cells with a knockdown of Cul4A (Figure 3i). Taken together, our results suggest that Cul4B-DDB1^DCAF8^ acts as an E3 ligase that regulates Cdc25A degradation.

### Cdc25A stability is negatively regulated by HDAC3-mediated deacetylation

**Figure 4:**
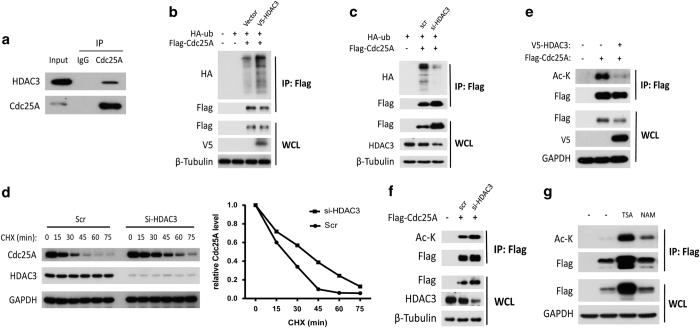
HDAC3 destabilizes Cdc25A through deacetylation**.** (**a**) Endogenous interaction between Cdc25A and HDAC3. IP was carried out using HEK293T lysates. (**b**) HDAC3 enhanced Cdc25A ubiquitination. HEK293T cells transfected with the indicated plasmids for 46 h were incubated with MG132 (10 μM) for 2 h, and were subjected to IP and western blot. (**c**) Knockdown of HDAC3 decreased Cdc25A ubiquitination. HEK293T cells transfected with HDAC3 siRNA for 24 h were transfected with the indicated plasmids for another 22 h, and were incubated with MG132 (10 μM) for 2 h, and then were lysed and subjected to IP and western blot. (**d**) HEK293T cells transfected with HDAC3 siRNAs for 48 h were treated with cycloheximide (CHX, 20 μg ml^−1^) for the indicated time, and were analyzed by western blot. (**e**) Overexpression of HDAC3 decreased the acetylation of Cdc25A. HEK293T cells transfected with the indicated plasmids for 48 h were lysed and analyzed by western blot or IP using anti-Flag antibody followed by western blot. (**f**) Knockdown of HDAC3 promoted the acetylation of Cdc25A. HEK293T cells treated with HDAC3 siRNA or plasmids for 48 h were lysed, and were analyzed by western blot or IP using anti-Flag antibody followed by western blot. (**g**) HEK293T cells transfected with Flag-Cdc25A for 38 h were treated with Trichostatin A (TSA, 5 μM) or nicotinamide (NAM, 5 mM) for 10 h, and were analyzed by western blot or IP using anti-Flag antibody followed by western blot.

In addition to phosphorylation and ubiquitination, acetylation has also been shown to be a major posttranslational modification that affects protein stability [30, 31]. As a deacetylase, HDAC3 was focused on, although acetylation has never been reported to regulate Cdc25A stability. As shown in Figure 4a and Supplementary Figure S4, the complex containing HDAC3 and Cdc25A was detectable at both endogenous and ectopic levels in cells. Overexpression and knockdown of HDAC3 enhanced and decreased Cdc25A ubiquitination, respectively (Figure 4b and c). Indeed, knockdown of HDAC3 significantly extended the half-life of endogenous Cdc25A (Figure 4d). Strikingly, the acetylation of Cdc25A was detected in cells, and this acetylation was diminished when the cells overexpressed HDAC3 (Figure 4e). In addition, both the acetylation and stability of Cdc25A were markedly increased in cells with a knockdown of HDAC3 and in cells treated with the HDACs inhibitor, Trichostatin A (Figure 4f and g).

### Cdc25A is stabilized by p300/CBP through the acetylation of lysine 150

**Figure 5:**
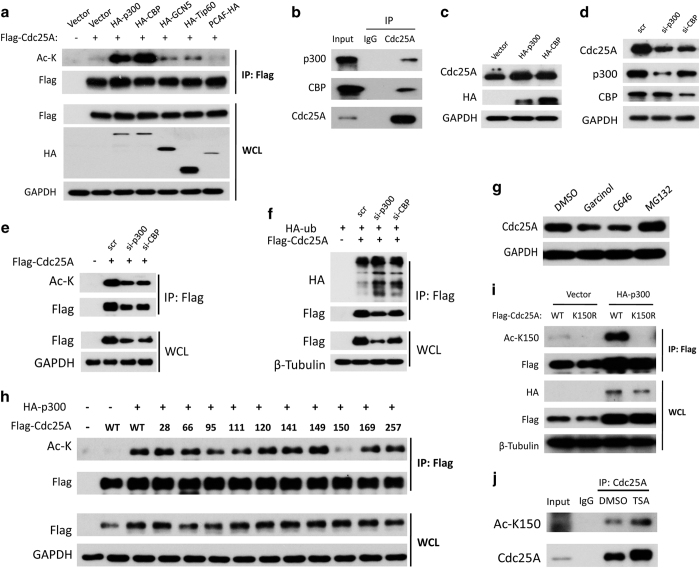
Cdc25A is stabilized by p300/CBP through acetylation of lysine 150**.** (**a**) p300/CBP acetylateed Cdc25A. HEK293T cells were co-transfected with Flag-Cdc25A and the indicated acetyltransferase plasmids for 48 h, and were analyzed by western blot. (**b**) Endogenous interaction between Cdc25A and p300/CBP. IP was carried out using HEK293T lysates. (**c**) Overexpression of p300/CBP stabilized Cdc25A. HEK293T cells transfected with HA-p300/CBP for 48 h were subjected to western blot. (**d**) Knockdown of p300/CBP destabilized Cdc25A. HEK293T cells transfected with siRNA targeting p300/CBP for 48 h were subjected to western blot. (**e**) Knockdown of p300/CBP decreased the acetylation of Cdc25A. HEK293T cells treated with the indicated siRNA or plasmids were lysed and analyzed by western blot or IP using anti-Flag antibody followed by western blot. (**f**) Knockdown of p300/CBP enhanced Cdc25A ubiquitination. HEK293T cells transfected with the indicated siRNAs or plasmids were analyzed by western blot or IP using anti-Flag antibody followed by western blot. (**g**) HEK293T cells were treated with Garcinol (10 μM), C646 (10 μM), or MG132 for 4 h, and were then analyzed by western blot. (**h**) Lysine 150 was the dominant acetylation site. HEK293T cells were co-transfected with p300 and Cdc25A mutated into arginine at the indicated lysine residue for 48 h, and were analyzed by western blot or IP using anti-Flag antibody followed by western blot. (**i**) Ac-K150 was detectable using a specific antibody. HEK293T cells were co-transfected with the indicated plasmids, and were analyzed by western blot or IP using anti-Flag antibody followed by western blot. (**j**) The acetylation of endogenous Cdc25A at K150 was detectable. HEK293T cells were treated with DMSO or Trichostatin A (TSA, 5 μM) for 10 h, and then were lysed and analyzed by western blot or IP using anti-Cdc25A antibody followed by western blot.

Next, we sought to identify the acetyltransferase responsible for acetylating Cdc25A. Among a series of tested acetyltransferases, both p300 and CBP interacted with and acetylated Cdc25A (Figure 5a and b, Supplementary Figure S5). Overexpression of p300 or CBP stabilized Cdc25A (Figure 5c), whereas knockdown of p300 or CBP not only decreased both the acetylation and the stability of Cdc25A (Figure 5d and e), but also enhanced Cdc25A ubiquitination (Figure 5f). Furthermore, Cdc25A was downregulated when cells were treated with Garcinol or C646, which are inhibitors of p300/CBP (Figure 5g). Collectively, these results reveal that the acetylation of Cdc25A, which is acetylated by p300/CBP and deacetylated by HDAC3, positively modulates its stability. To decipher the acetylation lysine (K) residue(s) within Cdc25A, three truncations of Cdc25A were generated. As shown in Supplementary Figure S6, the fragment of 1–170 amino acids may be the dominant region for acetylation. Then, each K in this fragment was separately mutated into an arginine (R). As shown in Figure 5h, K150 was identified as the dominant acetylation site. Indeed, as shown in Figure 5i and Supplementary Figure S7, the acetylation of K150 in exogenous Cdc25A was enhanced by p300 in both *in vitro* and *in vivo* assays using the ac-K150-specific antibody that we generated. More importantly, the K150 acetylation of Cdc25A was detectable at endogenous level using this specific antibody (Figure 5j).

### Acetylation of lysine 150 on Cdc25A delays its degradation and inhibits the G2/M checkpoint in response to ionizing radiation (IR)

**Figure 6:**
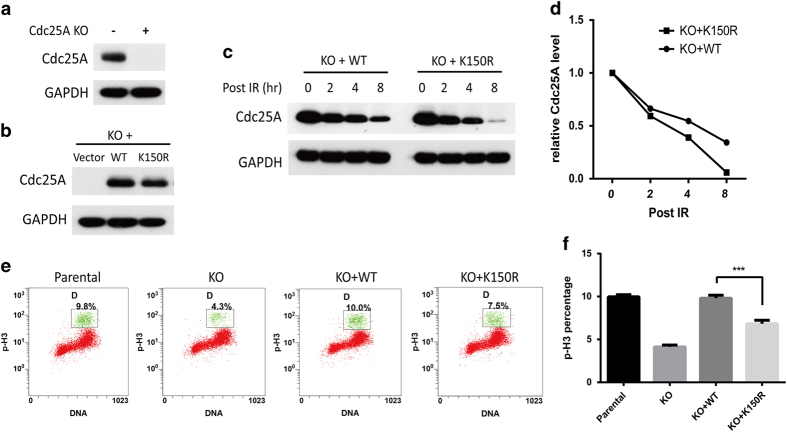
Acetylation of lysine 150 on Cdc25A negatively regulates the G2/M checkpoint in response to IR. (**a**) Knockout of endogenous Cdc25A in HeLa cells through CRISPR-Cas9. (**b**) Re-expression of wild-type (WT) or K150R mutant Cdc25A in the Cdc25A knockout cell line through pSIN lentivirus. (**c, d**) Degradation rate of WT-Cdc25A and K150R-Cdc25A mutant post IR (6 Gy) using western blot. (**e, f**) Cdc25A KO HeLa cells stably re-expressing WT-Cdc25A or K150R-Cdc25A mutant were exposed to IR (6 Gy). The cells were harvested and analyzed using flow cytometry after incubation with 100 ng ml^−1^ of nocodazole for 10 h. The percentage of cells positive for phospho-histone H3 (p-H3) is indicated. *N*=3. Bars indicate the s.e.m. ***P<0.001, Student’s *t-*test.

Given that Cdc25A is a labile protein during cell cycle and further destabilized under the DNA damage response [32], we sought to determine whether Cdc25A acetylation is also crucial for the response to IR. First, we knocked out Cdc25A in HeLa cells through the CRISPR-Cas9 system (Figure 6a). Second, we re-introduced wild-type Cdc25A (WT-Cdc25A) or the acetylation deficient mutant K150R (K150R-Cdc25A) in the Cdc25A knockout HeLa cells (Figure 6b). Compared with WT-Cdc25A, the K150R-Cdc25A mutant showed a faster degradation rate in response to IR (Figure 6c and d). More importantly, as shown in Figure 6e and f, knockout of Cdc25A strengthened the G2/M checkpoint in response to IR, which was shown by the decreased percentage of cells positive for phospho-Histone3, and this phenomenon was completely and only partially rescued by introducing WT-Cdc25A and the K150R-Cdc25A mutant into the cells with a knockout of Cdc25A, respectively. Statistically, WT-Cdc25A had a stronger capacity for inhibiting the G2/M checkpoint in response to IR than the K150R-Cdc25A mutant (Figure 6f), consistent with the result that WT-Cdc25A was more resistant to the IR-induced degradation than the K150R-Cdc25A mutant. Taken together, these results suggest that the acetylation of K150 on Cdc25A delays its degradation and has an inhibitory role on the G2/M checkpoint in response to DNA damage.

## Discussion

For a long time, a global understanding of the regulation of protein turnover has not been well explored because of the lack of an efficient method. In this report, we successfully established a system named Pro-SRSA that combines the CRISPR-Cas9 library, the pAd-DsRed-IRES-EGFP-X reporter and high-throughput sequencing to genetically screen for regulators of protein stability at a genome scale. In our Pro-SRSA, the low MOI of CRISPR-Cas9 library virus ensures the perturbation of one gene in each single cell, and the adenovirus-based infection avoids the disturbance of endogenous genes and could reach high infection efficiency without drug selection. In addition, the stability of protein X, which is reflected by the ratio of EGFP/DsRed, could be monitored through flow cytometry at the single-cell level, and the cells with a changed ratio induced by gene knockout would be sorted easily. Analyzing the enrichment of sgRNAs in the sorted cells through high-throughput sequencing would reveal the genes that regulate the stability of protein X.

**Figure 7:**
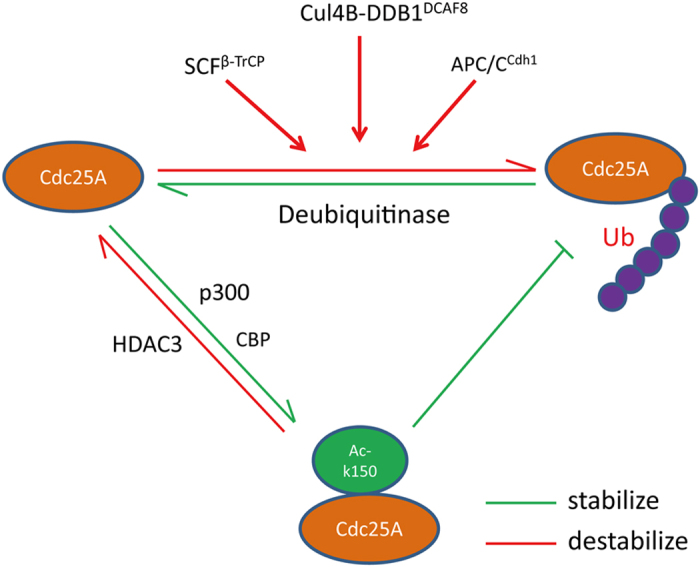
A proposed model for Cdc25A regulation in cells**.** Acetylation of Cdc25A at K150, which is acetylated by the acetyltransferase p300/CBP, and deacetylated by the deacetylase HDAC3, prevents its ubiquitin-mediated degradation by APC/C^Cdh1^, SCF^β-TrCP^ and Cul4B-DDB1^DCAF8^, and subsequently inhibits its functions, that is, the G2/M checkpoint in response to DNA damage.

Using our Pro-SRSA, we found that Cul4B-DDB1^DCAF8^ is an E3 ligase for the degradation of Cdc25A. This new E3 ligase, along with two well-established E3 ligases, APC/C^Cdh1^ and SCF^β-TrCP^ [26, 33], may coordinate with each other to closely regulate the Cdc25A level in different phases of the cell cycle and respond to a diversity of stresses (Figure 7). More interestingly, our Pro-SRSA has revealed that acetylation of Cdc25A at lysine 150, which is acetylated by p300/CBP and deacetylated by HDAC3, stabilizes its protein and delays its degradation in response to DNA damage (Figures 6 and 7). This unexpected finding indicates that acetylation, as a novel modification, along with multiple phosphorylations, affect Cdc25A ubiquitination and consequently regulate its stability. In fact, this acetylation-dependent regulation of Cdc25A turnover shows a high similarity with hSSB1 turnover, which has recently also been found to be regulated by acetylation [34]. These data demonstrate that acetylation modification may have a critical role in affecting protein homeostasis.

Recently, the CRISPR-Cas9 library has been demonstrated to be an efficient tool for performing genome-scale loss-of-function screening compared with a previous approach for screening that used a short hairpin RNA library, which is limited by the inherent incompleteness of protein depletion and confounding off-target effects [21, 35]. As the majority of the previous functional screenings using the CRISPR-Cas9 library were limited to proliferation-based phenotypes, such as drug resistance [20–23], our Pro-SRSA described here greatly extended the application of the CRISPR-Cas9 library by combining with fluorescence-based flow cytometric sorting. We speculate that the CRISPR-Cas9 library as a genetic tool could be more widely used through a combination with different detection systems such as a high-content imaging platform, mass spectrometry or a chemiluminescence system to explore biological processes and events. However, to achieve a high validation rate for such a screening, it is still necessary to improve the efficiency of a complete knockout and the consistency of distinct sgRNAs for the each gene, in addition to decreasing off-target effects. Fortunately, the GeCKO V2 library has recently been developed [36], which has better virus production and more sgRNAs per gene. We believe that the GeCKO V2 library would perform much better than the GeCKO V1 library using our Pro-SRSA. On the other hand, to improve the true discovery rate and minimize the false-positive rate, we strongly recommend performing multiple rounds of enrichment and at least two independent replicates for this screening. Notably, the diverse chromosomal copy number alterations of HeLa cells used in this study may impair the gene knockout efficiency, which in turn decrease the screening quality. Therefore, our Pro-SRSA for any protein would be better if using multiple cell lines.

In addition, our Pro-SRSA used the DsRed-IRES-EGFP-X reporter to monitor the protein degradation in live cells. However, for certain proteins, the N-terminal residues may undergo modifications and processing during or after translation, which may regulate localization, intracellular trafficking and even the stability of the protein [37]. In these cases, the C-terminal EGFP tag would be recommended as the reporter. On the other hand, the DsRed-IRES-EGFP-X reporter would ensure a certain ratio of EGFP/DsRed at a given time point. However, the activity of IRES may fluctuate as the IRES-transacting factors are susceptible to dynamic regulations. Moreover, translation of the gene downstream of an IRES may be less efficient than that of the upstream gene [38]. To avoid these shortages, the X-mEGFP-Ub_k0_-mRFP elements in the ProTA system may be a good reporter to be combined with the CRISPR-Cas9 library [19].

In summary, the Pro-SRSA will help us to better understand cellular protein regulation in a variety of physiological and/or pathological processes, which may open many avenues for disease curing. We suggest that this unbiased CRISPR-Cas9 screening method could be widely used to globally identify regulators of protein turnover.

## Materials and Methods

### Cells and reagents

HeLa, HEK293T and HEK293A cells were maintained in Dulbecco’s modified Eagle’s medium (DMEM; Life Technologies) supplemented with 10% fetal bovine serum (Life Technologies, Carlsbad, MA, USA) with 5% CO_2_ at 37 °C.

### Plasmids construction

The pCMV-DsRed-IRES-EGFP-X retroviral reporter vector was a gift of Professor Stephen J Elledge (Harvard University). DsRed-IRES-EGFP-X elements were subcloned into the pAd/PL-DEST vector (Life Technologies) to obtain the pAd-DsRed-IRES-EGFP-X adenoviral reporter vector. All of the transient ectopic expression vectors were constructed using the pCDNA3.1 vector (Invitrogen, Carlsbad, MA, USA). The pSIN lentivirus vector, which was inserted with Cdc25A, was used to generate stable cells overexpressing Cdc25A. The lenti-CRISPR plasmid was obtained from Addgene (Boston, MA, USA, cat. 49535, Feng Zhang’s Lab, MIT, Boston, MA, USA). The GeCKO lenti-CRISPR library was also obtained from Addgene (cat. 51241, Feng Zhang’s Lab).

### pAd-DsRed-IRES-EGFP-X adenovirus production

Cdc25A or p53 ORF was cloned into the pAd-DsRed-IRES-EGFP-X adenoviral reporter vector, *Pac*I digested and transfected into HEK293A cells cultured in 3.5 cm plates. Fourteen days later, the cells were harvested, and a crude viral lysate was prepared. The adenovirus was amplified by infecting 293A cells with the crude viral lysate. Two days later, the cells were collected and lysed by freezing and thawing three times, and then the cells were stored at −80 °C.

### Lenti-CRISPR library lentivirus production

HEK293T cells were seeded at ~50% confluence the day before transfection in DMEM supplemented with 10% fetal bovine serum. Transfection was performed using Lipofectamine 2000. For each 10 cm dish, 200 μl of Plus reagent was diluted in 1 ml of OptiMEM (Life Technologies) with 12 μg of lenti-CRISPR plasmid library, 4 μg of pVSVG and 8 μg of psPAX2. Then, 30 μl of Lipofectamine 2000 was diluted in 1 ml of OptiMEM, and after 5 min, this solution was added to the DNA mixture. The complete mixture was incubated for 20 min before being added to cells. After 6 h, the medium was changed to 12 ml of DMEM. After 48 h, the medium was removed and centrifuged at 3 000 r.p.m. at 4 °C for 10 min to pellet the cell debris. The supernatant was filtered through a 0.45 μm polyvinylidene difluoride membrane (Millipore, Darmstadt, Germany). The MOI was measured as described in Shalem *et al*. [21].

### Flow cytometry

To prepare the cells for flow cytometry sorting, live cells were harvested, resuspended in phosphate-buffered saline (PBS) with 10% fetal bovine serum and filtered using a 40 μm cell strainer (BD Falcon, Franklin Lakes, NJ, USA). Cell sorting was performed using a Beckman MoFlo Cell Sorting System (Beckman Coulter, Brea, CA, USA), and flow cytometry analysis was performed using a Beckman Cytomics FC500 Flow Cytometry System or a Gallios Flow Cytometry System (Brea, CA, USA).

### Lenti-CRISPR-Cas9 target sequences

E6AP: 5'-CCTAATCAGAACAGAGTCCC-3'; Cdh1-sg1: 5'-GTGGCATCGTGTTCTCATTC-3'; Cdh1-sg2: 5'-GCAGTACACGGAGCACCTGG-3'; Cdh1-sg3: 5'- TTCAGGTCACAGAGATGCGG-3'; DCAF8-sg1: 5'-CCACAGCGCTGGCGAGAACC-3'; DCAF8-sg2: 5'- CTCCTCGTCCGATGTGTCTG-3'; DCAF8-sg3: 5'-CTCATCAGAGTCCGCGTCTG-3'; DCAF8-sg4: 5'-GGCGCCGTGTACAGCGCAAG-3'; DCAF8-sg5: 5'-TAGCCCGCTTGCGCTGTACA-3'; DCAF8-sg6: 5'- TGCCGCGCTGGTTAAAGTGC-3'.

### Surveyor assay

The Lenti-CRISPR-Cas9 plasmid targeting E6AP was used to infect HeLa cells for 7 days. Then, genomic DNA was extracted from the cells, and 476-bp products containing the target sequence were amplified using the following primers: forward: 5'-AGCAGTAAGCATACTCTAACCAGTGA-3'; reverse: 5'-TGCTTATATGTGGAAGCCGGGTAAGA-3'. T7E1 (NEB, Ipswich, UK) digested as described in Shalem *et al*. [ 21].

### Genome-scale lenti-CRISPR infection and flow cytometry sorting procedure

First, 8×10^7^ HeLa cells were infected with the lenti-CRISPR library at 0.2 MOI (~250 cells per construct). One day later, the DMEM medium was changed to medium with puromycin selection (0.5 μg ml^−1^) for 7 days. Then, ten 10 cm dishes of library-infected HeLa cells were plated, and the cells were re-infected with the pAd-DsRed-IRES-EGFP-Cdc25A adenovirus at 4 MOI to ensure that >95% of the cells were positive. Two days later, the cells were trypsinized and resuspended in PBS. Then, cell sorting was performed using a Beckman MoFlo Cell Sorting System. As the cells were live, the 10 dishes of cells were not trypsinized together, the long sorting process may disturb the cell status and fluorescence. The best method for cell sorting is trypsinizing the cells in one dish and then performing sorting immediately. Performing trypsinization and sorting one dish at a time helps to maintain the stability of the cell status and fluorescence. We sorted the cells within the top 1% of the EGFP/DsRed ratio. In the screening, we prepared 10 individual 10 cm dishes, 8×10^6^ cells per dish, totally 8×10^7^ cells. For each dish, 5×10^6^ cells were really sorted and 3×10^6^ cells were left. Totally for 10 dishes, 5×10^7^ cells were sorted and 3×10^7^ cells were left. We used the purity priority mode, which discarded approximately 50% of the impure target cells. Finally, we obtained approximately 250 000 cells for analysis.

### sgRNA PCR amplification and high-throughput sequencing

Genomic DNA was extracted from 250 000 sorted cells and 1.3×10^7^ (approximately 200-fold more than the 64 751 sgRNAs) left unsorted cells using a TIANamp genomic DNA Kit (Tiangen, Beijing, China). Using the genomic DNA as a template, PCR was performed in two steps. The first step was performed to amplify the lenti-CRISPR sgRNAs from genomic DNA. The primers used are as follows: forward: 5'-TCTTGTGGAAAGGACGAAACACCG-3'; reverse: 5'-TCTACTATTCTTTCCCCTGCACTGT-3'. The second PCR was performed to attach Illumina adaptors and to barcode samples. The first-round amplicons were used as templates. The primers used are as follows: forward: 5'-AATGATACGGCGACCACCGAGATCTACACGTTCAGAGTTCTACAGTCCGACGATCttgtggaaaggacgaaacaccg-3'; reverse: 5'-CAAGCAGAAGACGGCATACGAGATTGCCGAGTGACTGGAGTTCCTTGGCACCCGAGAATTCCAtctactattctttcccctgcactgt-3'. Amplification was performed with 18 cycles for the first PCR and 25 cycles for the second PCR. The second-round amplicons were purified and sequenced using a HiSeq 2500 (Illumina, San Diego, CA, USA) with a single-end 100-bp run.

### Sequencing data processing

Sequencing data from HiSeq 2500 were analyzed and normalized as described in Shalem *et al*. [21].

### Establishment of Cdc25A knockout HeLa cell line

The sgRNA sequence, 5'- AAGAGCAGGCGGCGGCGGTG-3', which targeted exon of Cdc25A, was cloned into PX459 (Addgene), and the knockout cell line was generated as described in Shalem *et al*. [ 21].

### Western blot analysis

Cells were lysed in RIPA lysis buffer (50-mM Tris–HCl, 150-mM NaCl, 5-mM EDTA, 0.5% Nonidet P-40, and a protease and phosphatase inhibitor cocktail; Calbiochem, Darmstadt, Germany). Proteins were separated by SDS-PAGE and transferred to 0.45-μM polyvinylidene difluoride membranes (Millipore). The immunoblots were processed according to standard procedures using primary antibodies directed to EGFP, p21, GAPDH, HA, Flag, V5, HDAC3 (CST, Danvers, MA, USA), DsRed, Hsp70, p53, c-Myc, Cdc25A (Santa Cruz, Dallas, TX, USA), DDB1, Cul4A, Cul4B (Abcam, Cambridge, MA, USA), DCAF8 (Bethyl, Montgomery, TX, USA) and β-tubulin (Bioworld, Louis Park, China).

### Immunoprecipitation

HEK293T cells transfected with the indicated plasmids were lysed in RIPA lysis buffer and then centrifuged at 12 000 r.p.m. for 30 min. The supernatants were first incubated with anti-Myc-agarose (Santa Cruz), anti-FLAG-agarose (Sigma Chemical Co.) or anti-HA-agarose (Sigma Chemical Co., Darmstadt, Germany) overnight at 4 °C, and the precipitates were washed five times with RIPA lysis buffer.

### *In vitro* acetylation assay

Flag-Cdc25A and Flag-Cdc25A-K150R were synthesized using a PURExpress *In Vitro* Protein Synthesis Kit (NEB). Flag-Cdc25A and Flag-Cdc25A-K150R were incubated with p300 purified from HEK293T cells in HAT buffer (Millipore) in a 30 °C shaking incubator for 1 h. The effect of K150 acetylation was determined using western blot analysis.

### Generation of anti-Ac-K150-Cdc25A antibody

Rabbits were immunized with the coupled peptide FEFK-K(Ac)-PVRPV at Shanghai Genomics, Inc. (Shanghai, China) to generate anti-Ac-K150-Cdc25A, which is an antibody specific for acetylation on lysine 150 of Cdc25A.

### Phospho-histone H3 detection

Cells were exposed to IR (6 Gy) and incubated with 100 ng ml^−1^ of nocodazole for 10 h and were harvested and fixed in 70% ethanol at 20 °C. Then, cells were resuspended in 1 ml of 0.25% Triton X-100 in PBS, and incubated at 4 °C for 15 min with rocking. After the cells were centrifuged, the cell pellet was suspended in 100 ml of PBS containing 1% bovine serum albumin and 0.75 μg of a polyclonal antibody that specifically recognizes the phosphorylated form of histone H3 (Upstate Biotechnology, Lake Placid, NY, USA), and incubated for 1.5 h at room temperature. Then, the cells were rinsed with PBS containing 1% bovine serum albumin and incubated with fluorescein 488-conjugated goat anti-rabbit antibody (Jackson ImmunoResearch Laboratories, Boston, MA, USA) diluted at a ratio of 1:30 in PBS containing 1% bovine serum albumin. After the cells were incubated for 30 min at room temperature in the dark, they were stained with propidium iodide, and cellular fluorescence was measured using a FC 500 flow cytometer (Beckman Coulter).

## Figures and Tables

**Table 1:** A list of genes with high enrichment to be validated

*Number*	*Gene*	*Enrichment (fold)*
1	ZGPAT	111.87
2	STK11	48.22
3	CENPJ	26.67
4	MAP3K8	22.52
5	LIMK1	22.41
6	UBL4A	20.87
7	PPAP2A	20.69
8	WDR48	20.65
9	RAD23B	18.47
10	OCIAD2	17.50
11	PTCD1	13.91
12	USP47	13.84
13	RIOK2	13.52
14	DDB1	11.63
15	HDAC3	11.45
16	CAPN7	11.44
17	HES3	11.24
18	DCAF8	10.70
19	RNF13	10.43
20	KLK9	9.62

Abbreviations: sgRNA, single-guide RNA.The enrichment of each sgRNA, the numbers were calculated by dividing normalized sgRNA counts of sorted cells by those of unsorted cells.
