# Supplementary material for: A genome-scale CRISPR-Cas9 screening method for protein stability reveals novel regulators of Cdc25A
Source: Cell Discov. 2016 May 24;2:16014–. doi: 10.1038/celldisc.2016.14 (PMC4877570; doi:10.1038/celldisc.2016.14)
Supplement: Supplementary Figure S1 [file celldisc201614-s1.pdf]

Supplementary Figure 1. pAd adenovirus shows high infection efficiency.

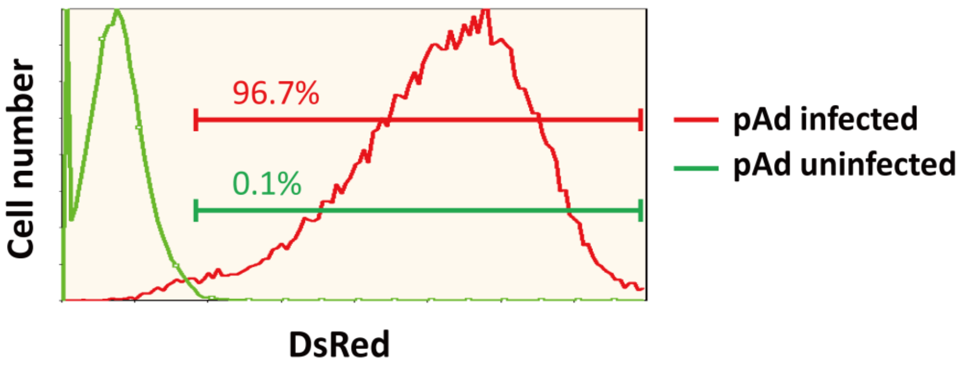

HeLa cells were infected with pAd-DsRed-IRES-EGFP-Cdc25A. Two days later, DsRed signal in the cells was detected by flow cytometry.
