# Supplementary material for: A genome-scale CRISPR-Cas9 screening method for protein stability reveals novel regulators of Cdc25A
Source: Cell Discov. 2016 May 24;2:16014–. doi: 10.1038/celldisc.2016.14 (PMC4877570; doi:10.1038/celldisc.2016.14)
Supplement: Supplementary Figure S2 [file celldisc201614-s2.pdf]

Supplementary Figure 2. Cell cycle distributions of the sorted and unsorted cells.

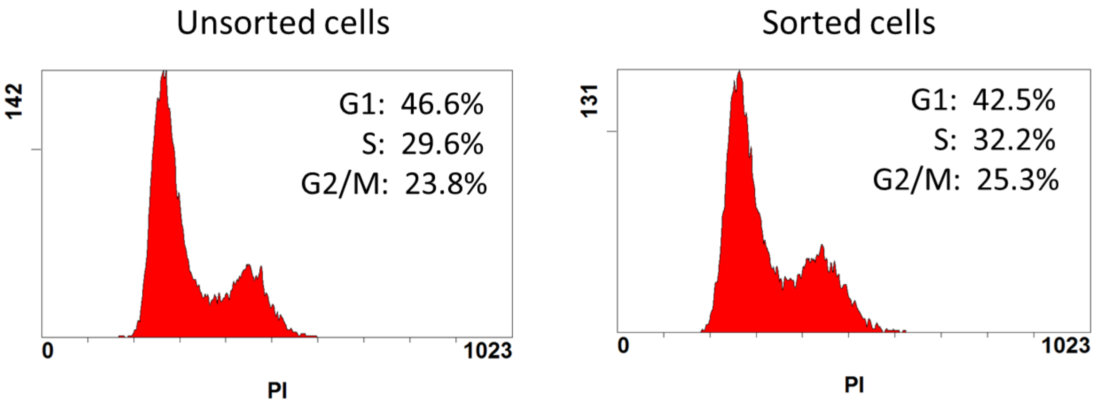

Both the sorted cells with high Cdc25A stability and unsorted cells were analyzed by PI staining and flow cytometry.
