# Supplementary material for: A genome-scale CRISPR-Cas9 screening method for protein stability reveals novel regulators of Cdc25A
Source: Cell Discov. 2016 May 24;2:16014–. doi: 10.1038/celldisc.2016.14 (PMC4877570; doi:10.1038/celldisc.2016.14)
Supplement: Supplementary Figure S3 [file celldisc201614-s3.pdf]

**Supplementary Figure 3. Exogenous interaction between Cdc25A and DDB1 or DCAF8.**

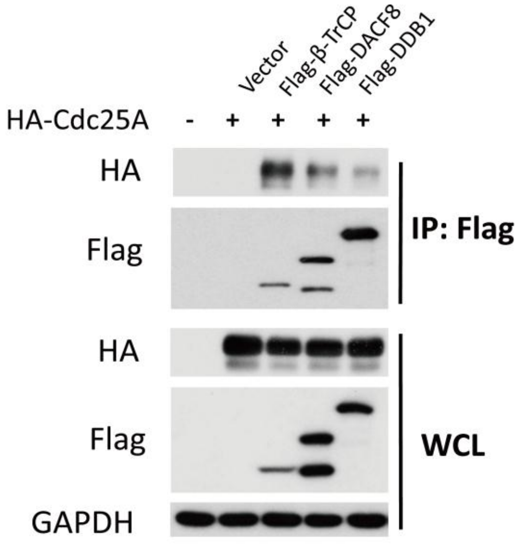


HEK293T cells transfected with the indicated plasmids for 48 hrs.were lysed with RIPA lysis buffer. Immunoprecipitation (IP) using anti-Flag antibody was performed, which was followed by Western blot analysis. β-TrCP was used as the positive control.
