# Supplementary material for: A genome-scale CRISPR-Cas9 screening method for protein stability reveals novel regulators of Cdc25A
Source: Cell Discov. 2016 May 24;2:16014–. doi: 10.1038/celldisc.2016.14 (PMC4877570; doi:10.1038/celldisc.2016.14)
Supplement: Supplementary Figure S6 [file celldisc201614-s6.pdf]

Supplementary Figure 6. 1-170 amino acids of Cdc25A is the dominant acetylation region.

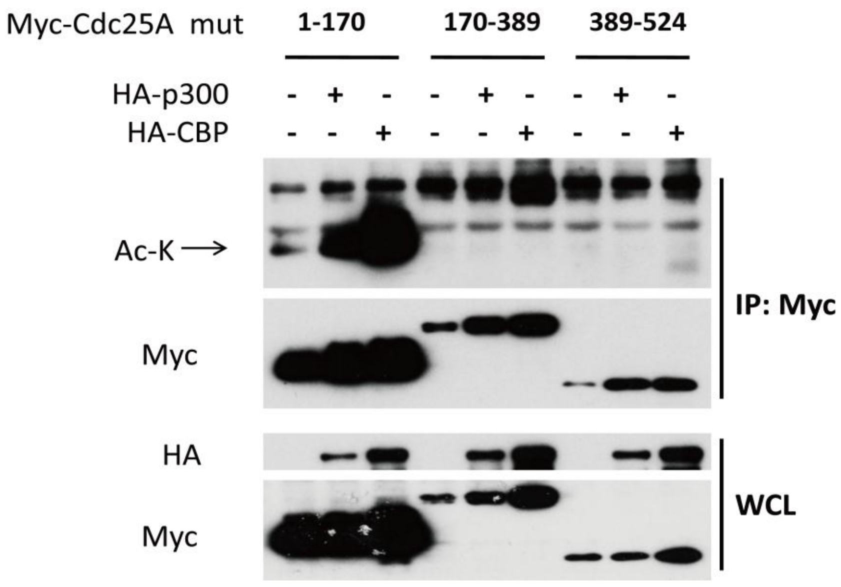

HEK293T cells co-transfected with the indicated truncation plasmids of Myc-Cdc25A, HA-p300 or HA-CBP for 48 hrs. were lysed with RIPA lysis buffer and subjected to IP using an anti-Myc antibody, which was followed by Western blot analysis.
