# Supplementary material for: A genome-scale CRISPR-Cas9 screening method for protein stability reveals novel regulators of Cdc25A
Source: Cell Discov. 2016 May 24;2:16014–. doi: 10.1038/celldisc.2016.14 (PMC4877570; doi:10.1038/celldisc.2016.14)
Supplement: Supplementary Figure S7 [file celldisc201614-s7.pdf]

**Supplementary Figure 7. p300 acetylates Cdc25A at K150 *in vitro.***

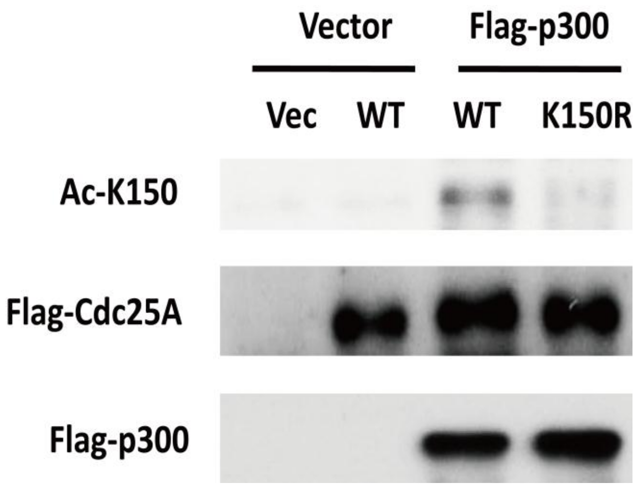


Flag-Cdc25A-WT or the Flag-Cdc25A-K150R mutant was incubated with p300 purified from HEK293T cells in acetylation buffer for 1 hr. *in vitro* and then analyzed using Western blotting.
